# Association between physical activity, sedentary time, and physical fitness of female college students in China

**DOI:** 10.1186/s12905-022-02108-y

**Published:** 2022-12-07

**Authors:** Ming Ming Guo, Xiao Zan Wang, Koon Teck Koh

**Affiliations:** 1 College of Physical Education and Health, East China Normal University, Shanghai, 200241 China; 2 Physical Education & Sports Science, National Institute of Education, Nanyang Technological University, Singapore, 637616 Singapore

**Keywords:** Female college students, Physical activity, Sedentary time, Physical fitness, Chinese

## Abstract

**Background:**

Prior research has highlighted the importance of Physical Activity (PA), Sedentary Time (ST), and Physical Fitness (PF) for health. However, there is limited research on the association between PA, ST, and PF in the context of female college students, particularly in Eastern cultures. Therefore, the purpose of this study was to investigate the association between PA, ST, and PF among Eastern female college students to inform policy and practice.

**Methods:**

The study was conducted from February to May 2022 at East China Normal University, Shanghai, China. A total of 512 Chinese female college students participated in this study through snowball sampling. Participants’ PA and ST were investigated using the International PA Questionnaire, and PF was measured using the Chinese National Student Physical Fitness Test. Independent samples t-test and binary logistic regression were used to compare the differences in PF between Active Participants (AP) and Inactive Participants (IP), as well as between Low Sedentary Participants (LSP) and High Sedentary Participants (HSP). Multinomial logistic regression was used to compare the differences in PF between Active and Low Sedentary Participants (ALSP), Active and High Sedentary Participants (AHSP), Inactive and Low Sedentary Participants (ILSP), and Inactive and High Sedentary Participants (IHSP). Data analysis was performed using Statistical Package for the Social Sciences version 28. The level of significance was set at 0.05.

**Results:**

AP showed significantly higher cardiorespiratory fitness (*p* < 0.05) and overall fitness (*p* < 0.01) than IP. Compared to IP, AP was less likely to be classified as unfit in terms of cardiorespiratory fitness [Odds Ratio (OR), 0.57; 95% Confidence Interval (CI), 0.38–0.85; *p* < 0.05]. Compared to IHSP, ALSP (OR, 0.44; 95% CI 0.25–0.76; *p* < 0.01) and AHSP (OR, 0.54; 95% CI 0.32–0.93; *p* < 0.05) were less likely to be classified as unfit in terms of cardiorespiratory fitness. Furthermore, compared to IHSP, AHSP (OR, 1.66; 95% CI 1.02–2.70; *p* < 0.05) and ILSP (OR, 2.09; 95% CI 1.16–3.77; *p* < 0.05) were more likely to be classified as unfit in terms of their flexibility.

**Conclusion:**

There was a positive association between PA, cardiorespiratory fitness, and overall fitness. There was also an association between cardiorespiratory fitness, flexibility, and the combination of PA and ST. These findings suggest that both PA and ST can influence different PF items. Therefore, we recommend that public health policy and practice for college female students should consider PF items when selecting different PA and ST intervention strategies.

## Background

Physical Activity (PA) refers to the energy expenditure behavior caused by any skeletal muscle movement in the human body [1]. Sedentary Behavior (SB) refers to sitting, lying, or planking during waking hours, which usually implies less energy expenditure [2]. Regular PA is a protective factor in preventing and controlling non-communicable diseases such as cardiovascular disease, type 2 diabetes, or types of cancers, and is also beneficial for one’s mental health and overall well-being [3]. Contrary to PA, high levels of SB are a risk factor for human health and all-cause mortality [3]. World Health Organization (WHO) characterized adults who do not complete 150 min of Moderate Physical Activity (MPA) or 75 min of Vigorous Physical Activity (VPA) per week as physically inactive [3]. A 2016 survey showed that 27.5% of adults worldwide are physically inactive [4]. Regarding SB, studies showed that most adults are sedentary for more than eight hours a day, and the trend has been rising [5]. In addition, the proportion of physically inactive females (31.7%) is significantly higher than that of males (23.4%) worldwide [4]. This trend is also observed at the college level. For example, Chinese female college students are significantly more sedentary than their male counterparts (7.63 h/day vs. 7.26 h/day) [6]. This trend appears to be present in Western contexts as well-American female college students appeared to be significantly less physically active than their male counterparts (2003 MET*mins/week vs. 2900 MET*mins/week) [7]. Hence, WHO has been working for decades to improve PA, reduce SB and increase equality in PA opportunities between genders and countries. Ultimately, WHO aims to reduce physical inactivity by 15% by 2030. However, the likelihood of achieving this goal is slim [3].

Physical fitness (PF) is a combination of cardiorespiratory fitness, body composition, muscular strength, muscular endurance, flexibility, balance, speed, and explosive power [1]. PF reflects an individual’s ability to perform daily PA or physical exercise. PF can also indicate a person’s current or future health status independent of PA. Several studies have shown that PF can predict the future probability of cardiovascular disease or all-cause mortality [8]. Better PF can improve the health of the skeletal muscular system [9], prevent a range of psychological disorders (e.g., depression, anxiety, psychological stress) [10], and reduce the risk of premature death [11]. A growing body of research has recently shown that PF is strongly associated with students’ cognitive and academic performance [12]. Given the importance of PF, most countries have established their PF measurements systems, such as the Fitness Gram in the United States [13], Eurofit in Europe [14], and the Chinese National Student Physical Fitness Test (CNSPFT) in China [15]. However, the results of these systems are worrying as they show that the PF of students at all levels had decreased over the past decade, accompanied by an increase in obesity, SB, and physical inactivity [16]. In China, this decline is particularly evident among college students. Research shows that some PF indicators for Chinese college students are lower than that of secondary school students [17], which is a worrying trend. The actual cause is unknown, and more research is warranted.

According to Blair’s theory, more PA leads to better PF [18]. And because PA, SB, and PF are all related to a person’s health status, it is intuitive that PA and PF indicators should be positively associated. The conventional wisdom is that higher PA and less Sedentary Time (ST) usually equates to better PF. However, recent studies have shown inconsistent results. For example, studies in Canada have shown that more PA is associated with better cardiorespiratory fitness, speed, and muscular strength [19]. Studies in the United States have shown that more PA and less ST tend to imply better cardiorespiratory fitness, muscular strength, speed, and flexibility [20, 21]. A study in Japan similarly showed a significant positive association between PA and overall fitness, cardiorespiratory fitness, speed, and muscular strength [22]. However, three studies conducted in the United States, Germany, and United Kingdom showed no association between PA [23, 24] or ST [25] and any indicator of PF. Such inconsistent findings are also reflected within China, such as two cross-sectional studies conducted in Beijing and Shandong Province, China, which showed that more ST tended to imply poorer speed, lower muscular strength, cardiorespiratory fitness, flexibility, and muscular endurance, while more PA indicated better muscular strength, overall fitness, and muscular endurance [26, 27]. A study conducted in Zhejiang Province, China, showed no association between ST and any PF indicators [28]. In addition, studies on the association between PA, ST, and PF among female college students are relatively scarce, especially in Eastern countries.

Considering these inconsistent findings from published studies to date, the significantly lower PA and PF levels of females than males, as well as the limited research available investigating a population of female college students who are influenced by Eastern culture and context, it is necessary to investigate the association between PA, ST, and PF among Eastern female college students to inform policy and practice.

## Materials and methods

### Study design and participants

A cross-sectional study was conducted from February to May 2022 at East China Normal University, Shanghai, China. A total of 512 first and second-year female college students participated in this study through a snowball sampling [18]. Healthy female students with no history of medical diseases and who were able to perform normal PA were included in the study. On the other hand, those with physical disabilities or illnesses were excluded from this study.

### Procedure

The protocol of this study was performed in accordance with the Declaration of Helsinki and approved by the Human Experimentation Ethics Committee of East China Normal University (Approval No. HR 330-2022). Participants were recruited by posting notices on campus and through online publicity. After recruitment, each student signed a consent form before participating in the study. They were told that participation in this study was voluntary and that they had the right to stop or withdraw from the study at any time. Subsequently, all participants were asked to complete an online questionnaire that included their names, contact information, physical education class information, and information on their PA and ST in the past week. After collecting participants’ data from the questionnaire, the second author of this study contacted the students’ physical education teachers and asked them to assist in administrating and collecting the PF data. The first author of this study participated in and supervised all data collection processes, ensuring the tests’ consistency. The data were subsequently sent to the first author for analysis.

### Physical activity measurements

Participants’ PA and ST were assessed using the Chinese version of the International Physical Activity Questionnaire Short Form (IPAQ-SF). The reliability and validity of this measurement tool have been validated in several previous studies and found to be suitable to measure PA and ST in Chinese college student populations [29, 30]. The IPAQ-SF measures participants’ PA and ST over seven days through seven questions. For the first six questions, participants were asked to fill in the number of days they performed VPA (e.g., high-intensity aerobic exercise), MPA (e.g., cycling), and Light Physical Activity (LPA, e.g., walking), and the approximate number of minutes per day they participated in each of these activities. The last question asked participants to fill in the average amount of ST (minutes) per day.

Data were converted, and outliers were removed before truncating the data according to the IPAQ guidelines [31]. ST, VPA, MPA, and MVPA were calculated separately using the following formula:

VPA = days of VPA * time of VPA per day (minutes), MPA = days of MPA * time of MPA per day (minutes), MVPA = VPA + MPA. ST = sedentary time per day (minutes).

Participants with MVPA > 150 min or VPA > 75 min over the past seven days were classified as the active group while the rest of the participants were classified as the inactive group based on WHO recommendations for adult PA [3]. The median of participants’ ST was used as the basis for grouping. This is in accordance with the ST grouping method proposed by Marques et al. [32]. Which categorized the group with longer ST as the high sedentary group and the group with shorter ST as the low sedentary group. In addition, participants were divided into four sub-groups, i.e., active/low sedentary group, active/high sedentary group, inactive/low sedentary group, and inactive/high sedentary group, based on the grouping of PA and ST.

### Physical fitness measurements

The PF of participants in this study was measured using the Chinese National Student Physical Fitness Test (CNSPFT), a test battery issued by the Chinese Ministry of Education [15]. The reliability of the CNSPFT has been validated (0.99), indicating that it can accurately measure the PF of Chinese college students [33]. There are seven items in CNSPFT: Body Mass Index (BMI, kg/m^2^), vital capacity (ml), 50-meter run (seconds), sit and reach (cm), standing long jump (m), one-minute sit-up (times), and 800-meter run (seconds), which represents body composition, cardiorespiratory fitness, speed, flexibility, muscular explosive, muscular endurance, and cardiorespiratory endurance of the subjects, respectively. The specific aim for each test and how to administer them correctly had been previously reported in another study[34]. Readers are encouraged to refer to it for more detailed information.

In terms of scoring, the results for each item were converted into a percentage score according to the scoring method specified in CNSPFT standards [15]. The overall fitness score was then calculated based on the score of each item using the following calculation formula from CNSPFT standards [15]:

Overall fitness score = body composition score*0.15 + cardiorespiratory score*0.15 + speed score*0.20 + flexibility score*0.10 + muscular explosive score*0.10 + muscular endurance score*0.10 + cardiorespiratory endurance score*0.20.

Each item score and the overall fitness score were classified into four levels: failing, passing, good, and excellent, according to the CNSPFT standards. Participants who achieved the excellent and good level for each item and overall fitness were categorized as the fit group for that item, and participants who did not achieve the excellent and good level (i.e., students with failing and passing levels) were categorized as the unfit group for that item.

### Statistical analyses

Participants with missing data were excluded from the study. Descriptive statistics of PA, ST, and each PF item were presented using mean ± standard deviation. Independent samples t-test was used to test whether there were any significant differences for the means of each PF item and the overall fitness between the active and inactive groups and between the high sedentary and low sedentary groups. Binary logistic regression was used to test the probability of the active group and the low sedentary group being classified as unfit on each PF item compared to the inactive group and the high sedentary group. Multinomial logistic regression was used to test the probability of the active/low sedentary group, active/high sedentary group, and inactive/low sedentary group being classified as unfit on each PF item compared to the inactive/high sedentary group. Data analysis was performed using Statistical Package for the Social Sciences (SPSS) version 28. The level of significance was set at 0.05.

## Results

After excluding data with missing PF indicators (11) or PA data that were out of range (6), a total of 495 participants were included in the final analysis. Table 1 presents the descriptive statistics for MVPA, ST, and each item’s result of PF. Overall, participants spent 233.35 ± 219.63 min per week in MVPA and 431.52 ± 183.75 min per day in ST, with 62% (n = 305) of participants meeting the WHO PA guidelines (a total of at least 150 min of MVPA or at least 75 min of VPA per week) [3]. The active group had significantly higher cardiorespiratory fitness (vital capacity; *p* < 0.05) and overall fitness (*p* < 0.01) than the inactive group. In the end, 43% (n = 213) of participants who were sedentary for less than the median ST (420 min per day) were categorized as the low sedentary group. There was no significant difference between the low and high sedentary groups for each item of PF.

**Table 1:** Descriptive statistics of participants’ physical activity, sedentary time, and physical fitness

Variable	All (495)	Active (305)	Inactive (190)	*p*	Low sedentary (213)	High sedentary (282)	*p*
PA/ST							
MVPA (mins/week)	233.35 ± 219.63	333.61 ± 225.25	72.42 ± 47.08	0.000**	254.37 ± 218.31	217.48 ± 219.67	0.060
ST (mins/day)	431.52 ± 183.75	425.02 ± 182.76	441.95 ± 185.35	0.319	256.69 ± 96.95	563.56 ± 107.71	0.000**
Fitness							
BMI (kg/m^2^)	20.02 ± 1.77	20.11 ± 1.83	19.88 ± 1.68	0.161	20.07 ± 1.67	19.99 ± 1.85	0.621
Vital capacity (ml)	2819.18 ± 426.16	2857.10 ± 438.66	2758.31 ± 398.92	0.012*	2861.91 ± 405.43	2786.91 ± 439.13	0.052
800 m (seconds)	213.78 ± 18.4	213.43 ± 18.52	214.35 ± 18.26	0.591	213.29 ± 18.94	214.16 ± 18.01	0.605
Sit-up (times)	45.77 ± 8.58	46.27 ± 8.89	44.97 ± 8.02	0.103	45.22 ± 8.22	46.19 ± 8.84	0.215
Sit and reach (cm)	19.72 ± 5.45	19.74 ± 5.54	19.68 ± 5.31	0.900	19.65 ± 5.28	19.77 ± 5.57	0.806
Standing long jump (m)	1.79 ± 0.14	1.79 ± 0.14	1.79 ± 0.14	0.874	1.77 ± 0.13	1.81 ± 0.14	0.080
50 m run (seconds)	8.33 ± 0.46	8.34 ± 0.46	8.31 ± 0.47	0.459	8.31 ± 0.48	8.34 ± 0.45	0.519
Overall fitness	83.48 ± 2.93	83.74 ± 3.17	83.05 ± 2.44	0.007**	83.38 ± 2.82	83.55 ± 3.02	0.525

Participants were classified as a low sedentary group and a high sedentary group according to the median of participants’ ST, which is 420 minutes per day

All tests of variance were performed using independent samples t-tests

All indicators are presented as mean ± standard deviation

Bold values denote statistical significance at the *p* < 0.05 level

*indicates *p* < 0.05, **indicates *p* < 0.01

Table 2 presents the association between PA, ST, and each item of PF. Compared with the inactive group, the active group was less likely to be classified as unfit in terms of cardiorespiratory fitness (vital capacity; Odds Ratio (OR), 0.57; 95% Confidence Interval (CI), 0.38–0.84; *p* < 0.01). After adjusting for the effect of ST, the active group was still significantly less likely to be classified as unfit than the inactive group in cardiorespiratory fitness (vital capacity; OR, 0. 57; 95% CI 0.38–0.85; *p* < 0.05). This result indicates a positive association between PA and cardiorespiratory fitness.

**Table 2:** Association between physical fitness classification with physical activity and sedentary time groups (n = 495)

	PA group (using the inactive group as reference)		ST group (using the high sedentary group as reference)	
	Active	Active^a^	Low sedentary	Low sedentary^a^
BMI				
Fit	1.00	1.00	1.00	1.00
Unfit	1.35 (0.54–3.38)	1.39 (0.55–3.47)	0.61 (0.24–1.51)	0.60 (0.24–1.49)
Vital capacity				
Fit	1.00	1.00	1.00	1.00
Unfit	0.57 (0.38–0.84)**	0.57 (0.38–0.85)*	0.754 (0.518–1.10)	0.77 (0.53–1.12)
800 m				
Fit	1.00	1.00	1.00	1.00
Unfit	0.72 (0.49–1.07)	0.72 (0.49–1.07)	1.09 (0.74–1.61)	1.11 (0.75–1.64)
Sit-up				
Fit	1.00	1.00	1.00	1.00
Unfit	0.82 (0.57–1.18)	0.81 (0.57–1.17)	1.11 (0.78–1.58)	1.12 (0.78–1.60)
Sit and reach				
Fit	1.00	1.00	1.00	1.00
Unfit	0.93 (0.65–1.34)	0.93 (0.65–1.35)	0.888 (0.62–1.27)	0.89 (0.62–1.28)
Standing long jump				
Fit	1.00	1.00	1.00	1.00
Unfit	0.92 (0.64–1.32)	0.90 (0.63–1.30)	1.37 (0.96–1.96)	1.38 (0.96–1.97)
50 m run				
Fit	1.00	1.00	1.00	1.00
Unfit	1.02 (0.71–1.47)	1.03 (0.72–1.48)	0.91 (0.64–1.30)	0.91 (0.63–1.29)
Overall fitness				
Fit	1.00	1.00	1.00	1.00
Unfit	0.42 (0.14–1.27)	0.42 (0.14–1.28)	0.83 (0.33–2.09)	0.86 (0.34–2.17)

Data are OR and 95% CI

^a^ PA is additionally adjusted for ST, and ST is mutually adjusted for PA

Bold values denote statistical significance at the *p* < 0.05 level

*indicates *p* < 0.05, **indicates *p* < 0.01

Table 3 presents the association between the combination of PA, ST, and each item of PF. Compared with the inactive/high sedentary group, the active/low sedentary group (OR, 0.44; 95% CI 0.25–0.76; *p* < 0.01) and the active/high sedentary group (OR, 0.54; 95% CI 0.32–0.93; *p* < 0.05) was less likely to be classified as unfit in terms of cardiorespiratory fitness (vital capacity), indicating that the combination of PA and ST was associated with cardiorespiratory fitness (vital capacity). Furthermore, compared to the active/low sedentary group, the active/high sedentary group had an increased probability of being classified as unfit in terms of cardiorespiratory fitness (vital capacity), demonstrating that besides being active, less ST could further reduce the probability of participants being classified as unfit in terms of cardiorespiratory fitness (vital capacity). In addition, compared with the inactive/high sedentary group, the active/high sedentary group (OR, 1.66; 95% CI 1.02–2.70; *p* < 0.05) and the inactive/low sedentary group (OR, 2.09; 95% CI 1.16–3.77; *p* < 0.05) were more likely to be classified as unfit in terms of flexibility (sit and reach), indicating that the combination of PA and ST was also associated with the flexibility (sit and reach). Furthermore, compared to the inactive/high sedentary group (probability, 1), the inactive/low sedentary group (probability, 2.09) and the active/high sedentary group (probability, 1.66) had an increased probability of being classified as unfit in terms of flexibility (sit and reach), indicating that high sedentary with more PA, as well as inactive with less ST are associated with worse flexibility.

**Table 3:** Association between physical fitness classification with the combination of physical activity and sedentary time (n = 495)

	PA/sedentary time group (using the inactive/high sedentary group as reference)		
	Active/low sedentary	Active/high sedentary	Inactive/low sedentary
BMI			
Fit	1.00	1.00	1.00
Unfit	0.82 (0.26–2.63)	1.02 (0.35–2.95)	0.24 (0.03–2.04)
Vital capacity			
Fit	1.00	1.00	1.00
Unfit	0.44 (0.25–0.76)**	0.54 (0.32–0.93)*	0.72 (0.37–1.37)
800 m			
Fit	1.00	1.00	1.00
Unfit	0.79 (0.47–1.35)	0.64 (0.38–1.08)	0.94 (0.51–1.74)
Sit-up			
Fit	1.00	1.00	1.00
Unfit	0.92 (0.56–1.51)	0.89 (0.55–1.43)	1.28 (0.71–2.30)
Sit and reach			
Fit	1.00	1.00	1.00
Unfit	0.87 (0.52–1.47)	1.66 (1.02–2.70)*	2.09 (1.16–3.77)*
Standing long jump			
Fit	1.00	1.00	1.00
Unfit	1.24 (0.75–2.04)	0.88 (0.55–1.41)	1.33(0.74–2.38)
50 m run			
Fit	1.00	1.00	1.00
Unfit	0.91 (0.56–1.50)	0.858 (0.533–1.381)	0.691 (0.385–1.238)
Overall fitness			
Fit	1.00	1.00	1.00
Unfit	0.44 (0.11–1.68)	0.62 (0.16–2.46)	2.03(0.21–19.86)

Data are OR and 95% CI

Bold values denote statistical significance at the *p* < 0.05 level. *indicates *p* < 0.05, **indicates *p* < 0.01

## Discussion

The purpose of this study was to examine the association between PA, ST, and PF in Chinese female college students. Our results showed that 62% of the participants met the WHO guidelines for PA in adults. We found that higher PA was associated with better cardiorespiratory fitness and overall fitness. Furthermore, more PA with less ST was associated with better cardiorespiratory fitness and poorer flexibility. These findings are partially consistent with previous studies on the association of PA, SB, and PF around the world [3, 6, 9], but we also found some interesting results that contradict previous studies.

Unlike the PA guidelines for children and adolescents, which require at least 60 min of MVPA per day, the WHO guidelines for adults only require a minimum of 150 min of MVPA or 75 min of VPA per week [3]. Only 62% of the participants in this study met this requirement, which is generally consistent with high-income countries in the Asia-Pacific region (64%) and Western high-income counties (63%) but significantly lower than East Asian countries (83%) like China [4]. The reasons could be attributed to Chinese college students having less PA related to work, housework, or commuting than working adults. In addition, in the traditional Chinese culture, girls are expected to remain quiet and well-behaved rather than outspoken or physically active [35]. According to Bandura’s social cognitive theory, environment and culture are important factors influencing how people think and behave [36]. Indeed, a study in Iran showed inconsistency in emotional intelligence between male and female college students due to cultural and environmental influence [37]. We argue that specific cultural and social contexts in China can shape the attitudes, habits, and behaviors of female college students who perceive and are involved in PA, ST, and PF differently than in Western culture. Indeed, recent cultural comparative research showed that the majority of the sports programs designed to teach values in Western countries are not always successfully implemented in Asian contexts due to cultural differences in the way students learn and practice as well as what values are deemed important to them [38]. A good starting point for future study could be to understand female college students’ values and belief systems in PA participation and how they frame their judgments and decision-making.

The overall fitness is a composite representation of the participants’ PF levels. This study showed that the overall fitness of the active group was significantly higher than that of the inactive group. The result is consistent with previous studies that examined the association between PA and PF in high-income countries such as Portugal [32] and low-middle-income countries such as Iran [39]. Hence, the present study’s results further validated the importance of PA for college students. Consistent with some earlier studies on the association between ST and PF in high-income countries [32] and China [28], this study’s result also showed no significant difference between the low sedentary group and the high sedentary group in all PF items. The result confirmed Blair’s theory that more PA leads to better PF rather than sedentary behavior [18]. We argue that PA leads to better PF probably because the PA of college female students includes an exercise component such as running and swimming. However, physical activities such as walking, cleaning, and ST do not have any effect on the overall fitness of the students. To improve the overall fitness of female students, the college should start by improving their exercise time.

Vital capacity is the volume of air exhaled to the best of one’s ability after a maximum inspiration and represents the participants’ cardiorespiratory fitness [40]. The active group in the present study had significantly higher cardiorespiratory fitness than the inactive group. They also had a lower probability of being classified as unfit in the active group compared to the inactive group regarding cardiorespiratory fitness, implying a positive association between PA and cardiorespiratory fitness. This result is consistent with previous studies on the association between PA and cardiorespiratory fitness in high-income countries such as Canada [41] and low-middle-income countries such as South Africa [42]. Previous studies often used long-distance running as a proxy for participants’ cardiorespiratory fitness and have found a positive association between PA and long-distance running such as a 1-mile run [43] or a 20-meter shuttle run [41]. However, it should be noted that the 800-meter run, another proxy for cardiorespiratory fitness used in this study, did not show any association with PA. One explanation for this phenomenon is that the 800-meter run test may not be sensitive enough to detect participants’ cardiorespiratory fitness adequately as compared to the longer distance (e.g., 1 mile) tests in other studies [44]. More research is warranted to validate this observation. The results of multinomial regression in this study showed that the combination of PA and ST was also associated with cardiorespiratory fitness. Being active with less ST could further reduce the probability of participants being classified as unfit in cardiorespiratory fitness. We believe that the reason for this result is that ST will counteract some of the benefits of PA for cardiorespiratory fitness. Therefore, health promotion agencies and organizations should include strategies such as the promotion of PA while reducing ST to improve the cardiorespiratory fitness of college female students.

Sit and reach is a movement in which the subject sits on a flat surface with their legs in a straight position before bending over and stretching their hands toward their feet. It represents the flexibility of the subject’s back and legs [45]. Interesting findings were found in this study regarding the association between participants’ flexibility and the combination of PA and ST. Specifically, compared to the inactive/high sedentary group, the inactive/low sedentary group and the active/high sedentary group were more likely to be classified as unfit in terms of flexibility, implying less ST and more PA were associated with worse flexibility. These results are contrary to the findings of previous studies, which showed that more PA or less ST equates to better flexibility in both the United States [46] and Brazil [47]. One possibility for this phenomenon is that the active/high sedentary group and the inactive/low sedentary group had become aware of their worse flexibility and begun to increase their PA or reduce their ST to improve their flexibility. Hence, their flexibility will likely continue to be lower than the inactive/high sedentary group for some time. The benefits of more PA may be offset by more ST and vice versa [48], so it is reasonable to assume that more PA or less ST for female college students with high ST or low PA will not contribute much to improving their flexibility, and may even lead to a worsening of the pre-existing state (i.e., decrease in flexibility). Further studies on the association between PA, ST, and flexibility are warranted to advance our knowledge.

BMI, sit-up, standing long jump, and the 50-meter run represent the participants’ body composition, muscular endurance, explosive power, and speed, respectively. The present study showed no significant association between PA, ST and body composition, muscular endurance, explosive power, and speed. These results are inconsistent with previous findings in several high-income countries and China, which showed a positive association between PA and body composition [49], muscular endurance [50], and speed [21]. A negative association was observed between ST and body composition [51], explosive power [52], and speed [26]. However, such findings are consistent with previous studies in the United States and Brazil [21, 53]. According to Rhodes [54] and Netz [55], people’s self-efficacy and beliefs about PA differed significantly by age and gender. For example, a female’s psychological state varies with age [56]. Therefore, a possible explanation might be that the association between PA, ST, and the above items existed only in specific age groups or genders. Indeed, a few studies found an association between PA, ST, body composition, muscular endurance, explosive power, and speed involved children and adolescents [49–52]. However, other studies showed no association between PA, and ST, and those items mentioned in the present study were generally conducted on females [53] or adults who were in the same population as the present study [21]. Therefore, we believe that the association between PA, ST, body composition, muscular endurance, explosive power, and speed is more likely to be found in studies involving children and adolescents or mixed-gender students and that the association should be insignificant or non-existent for female Chinese college students. In light of these results, we suggest that gender and age should be taken into account when conducting PA and ST-based initiatives to improve students’ body composition, explosive power, muscular endurance, and speed.

To our knowledge, the present study was the first to examine the association between PA, ST, and PF among female Chinese college students. The findings of this study provided helpful information for the government or college administrators to develop health promotion policies or programs for female college students. The strength of this study was that the sample was drawn from many female college students from an underrepresented population in the literature. Also, this study analyzed the association between each PF item and PA or ST.

Despite these contributions, this study has several limitations that are worth mentioning. Firstly, the samples were selected from the same university in Shanghai, China, which may not be a good representation of all female college students in China. Future studies should include college participants from other provinces. Secondly, most participants were either first- or second-year students, with few third- or fourth-year students. Thus, the results did not sufficiently represent all college student’s grades in China. Future studies should include more third- and fourth-year college students to address this gap to form a more representative sample. Thirdly, participants’ PA was not measured by objective measurement devices such as the triaxial accelerometers but by a subjective questionnaire, which may be subject to recall bias. Future studies should consider utilizing the objective accelerometer to address this gap. Finally, the association between various indicators of PF, multiple types of PA (e.g., work, housework, transportation, etc.), and ST (e.g., study, screen time, etc.) should be further explored as these may be confounding factors that affect the overall results.

## Conclusion

A positive association between PA, cardiorespiratory fitness, and overall physical fitness was observed in our study. In addition, the results of this study also suggested that more PA and less ST are associated with better cardiorespiratory fitness and worse flexibility. These findings suggested that both PA and ST can influence different PF items of Chinese female college students. From the perspective of public health and health education, these results re-emphasized the critical and different roles that PA and ST play while shedding light on the need for health promotion actions or health education to be targeted according to gender or age.

## Data Availability

The datasets analyzed in the current study are available from the corresponding author upon request.
